# Operating room nurses’ experiences of limited access to daylight in the workplace – a qualitative interview study

**DOI:** 10.1186/s12912-021-00751-8

**Published:** 2021-11-10

**Authors:** Jenny Golvani, Linda Roos, Maria Henricson

**Affiliations:** 1 Surgical Operation and Intensive Care Unit, Ryhov County Hospital, Box 1024, 551 11 Jönköping, Sweden; 2 Surgical Operation and Intensive Care Unit, Höglandssjukhuset, Västanåsgatan 9, 575 81 Eksjö, Sweden; 3 Department of Nursing Science, IMPROVE-research Group, School of Health and Welfare, Jönköping University, S-551 11 Jönköping, Sweden

**Keywords:** Daylight, Experiences, Focus group, Operating room nurse, Qualitative, Well-being, Work environment

## Abstract

**Background:**

The operating room nurse is, among other things, responsible for patient safety and maintaining an aseptic environment. For hygienic reasons unnecessary traffic in the operating room should be avoided, which may mean that the operating room nurse works long shifts without relief. Operating departments are usually separated, where there might be no daylight opportunities in the operating room. The purpose of the study was to describe operating room nurses’ experiences of limited access to daylight in the workplace.

**Method:**

Qualitative design with four semi-structured focus groups of totally 15 operating room nurses. The analysis was performed with a content analysis with an inductive approach.

**Results:**

The study generated two main categories, difference in light and contact with the outer world. Operating room nurses felt that daylight affected them differently from the light from lamps, where daylight was considered important for experiencing well-being. Daylight could lead to a sensation of joy but also increased awareness and energy which seemed to improve the ability to perform at work. The limited access to daylight contributed to fatigue and led to an internal stress that affected the nurses even after work. Having opportunities to look out through windows under a workday was important to experience contact with the outside world and created a sense of time.

**Conclusion:**

To look out can reduce the feeling of being trapped in the closed context that the operating department entails. It can also lead to increased well-being and comfort in the workplace. We consider that daylight is an important component in the physical work environment that needs to be taken into consideration in further research as well as in new construction of operations departments.

## Introduction

Surgery wards are often detached, lacking windows and daylight [1]. Operating room nurses rarely leave the operating room before the surgery is done and the patient is awake and ready to be transported to postoperative care; this is to maintain patient safety and an aseptic environment [1, 2]. This situation can result in the absence of daylight for a significant portion of the workday for the operating room nurse. Incoming daylight provides information regarding the time of day and supports the human circadian rhythm, and it is therefore suggested that permanent workplaces have access to daylight.

Surgical wards are technologically advanced, and the operating room nurse is responsible for maintaining patient safety and an aseptic environment in the surgical field [3]. Working in shifts with long working hours are part of the operating room nurses work environment [4] with a 30 min lunch break and two shorter breaks for coffee during a dayshift [1]. These breaks are rarely used as they pose a risk to an aseptic environment where the number of people in an operating room going in and out should be kept at a minimum. A disease can spread through the air or through direct contact which forces the routines of the surgical environment to minimize traffic [2]. The surgical outcome is influenced by the physical and psychological environment as well as organisational components in the workplace.

People working indoors are usually exposed illuminance levels of 200–500 lx in a combination of natural and electrical light [5]. The recommended light levels for the surgical environment are 1000 lx as general lighting and 10,000–100,000 lx over the surgical field [6]. Artificial light has previously been shown to contribute to fatigue and headache, and electrical light can decrease sight [5]. Both artificial light and daylight can cause visual discomfort, either directly via the light sources or indirectly via reflections in the surrounding environment [5, 7]. To decrease glare and the subsequent decrease in work efficiency, the visual environment of the surgical environment needs to meet visual and ergonomic standards [6].

Lack of exposure to daylight is a known factor for hormonal imbalance such as vitamin D deficiency [8]. The human circadian rhythm (response to light and dark) regulates sleep, food intake, hormonal levels, blood pressure and body temperature [8]. Lack of light or light exposure at the wrong time of the day has proven to result in disturbances of circadian rhythm [9, 10]. Additional effects beyond vision include mood changes as well as cognitive dysfunction or depression [10]. According to Mead [11] the production of serotonin is affected by exposure of daylight and is converted to melatonin at night. Melatonin creates sleepiness and the production is controlled by exposure of light and dark. Working indoors with a limited exposure to daylight disturbs the production of melatonin. Low levels of serotonin results in a later production of the nightly melatonin [11, 12]. Symptoms such as decreased activity, increased need of sleep, weight gain and increased appetite are experienced [12, 13].

In a literary review, Aries et al. [7] showed that people with the ability to work close to a window indoors appreciated their work to a higher extent. The natural light changes during the day, and even the changes in weather showed to affect the mood and health of the individual. Lack of ventilation and lighting affects a person, as they feel more tired, resulting in an experience of difficulty to perform at their workplace [14]. Health care workers desire access to daylight in order to prevent fatigue during the workday [15]. Fatigue in nurses increases the risk for mistakes as well as an inability to discover changes in the health status of a patient. Fatigue could also result in a decreased perception of one’s own health and wellbeing at work, as well as difficulties maintaining their social interactions [16]. Existing research on the effects of daylight on the wellbeing on health care workers is scarce [7, 17]. Low exposure to daylight on surgical and/or intensive care wards have been associated with stress and decreased contentment at work [18] and have the opposite effect at exposure to daylight [19]. Certain surgical wards have less access to daylight compared to other wards in the hospital. The surgical context is therefore a suitable setting in which to study the operating room nurses’ experience of wellbeing when exposure to daylight is limited [19].

The surgical context is complex, including many factors affecting staff as well as patients. In order to minimize traffic in and out of an operating room the operating room nurse may have to remain in the room for extended periods of time without the option of leaving. Absence of daylight has proven to affect a person’s performance negatively. Lack of daylight can also affect the circadian rhythm resulting in cognitive effects. To be able to manage a good health care system it is necessary to ensure that the environment in which the staff works contributes to wellbeing and health.

## Method

### Aim

The aim of the study was to describe operating room nurses’ experiences of limited access to daylight in the workplace.

### Design

A qualitative explorative design with focus group interviews was used to obtain a detailed understanding how nurses experience their working environments with limited access to daylight [20]. This study complied with the consolidated criteria for reporting qualitative research (COREQ) checklist.

### Setting and participants

Using an availability approach [20], a surgical ward at a county hospital in the south of Sweden was selected. The head of the department was contacted in order to obtain permission to execute the study. The surgical ward at the hospital is located at the underground level, where “light courts” allow light to reach certain parts of the workplace. Windows are present in staff areas as well as a few corridors, whereas no windows are found in any of the operating rooms. The break room and the corridor outside the bathrooms had the most access to windows. The informants were chosen by the operating coordinator according to access to staff. The inclusion criteria for the study were: Operating room nurses working more than 50% clinically at the surgical ward. The unit manager sent an email to all (*n* = 30, one male and 29 female) operating room nurses at the ward. A total of 15 nurses was included in the study. All included operating room nurses were working both day and night shifts different specialized fields within elective surgery. Four focus groups interviews were carried out with 3–4 female participants per group, of which one participant declined. The informants were female, 27–63 years (mean 44,5 years), with a working experience between 1 and 40 years (mean 14,5).

### Data collection

To reach the aim of the study, focus group interviews with semi-structured questions was used [21]. The questions were arranged from general to specific and completed with follow up questions (see Table 1) [20]. To maintain the result’s credibility, a test interview was performed where the questions were tested [21], and one question was rephrased. The test interview was not included in the study.

**Table 1:** Interview guide

**Theme 1**	- What does light and daylight mean to you?
**Theme 2**	- What is your experience of the environment in the operating theatre and the lack of daylight
**Theme 3**	- What is your experience of the environment in the operating theatre and the access to daylight?
**Theme 4**	- What is your experience regarding limited access to daylight during a workday? How does it affect you? - What do you do to access daylight during a workday?
**Theme 5**	- What changes would you want to see in the operating theatre that would improve the working environment?

The interviews were performed in February 2020, in a room chosen by the head of the unit, in close access to the surgical ward. During the focus group interviews, the first and second author took turns on moderating and observing the interview. The moderator asked the questions and made sure every participant was given a chance to speak and decided when it was time to move on to the next question. The observer was located at a chair next to the table with the informants and in addition to the recording, she kept notes during the interviews. The interviews were recorded and lasted between 30 and 60 min per group. The interviews were transcribed, including notes regarding facial expressions, body language, sighs, and tone of voice in order to facilitate the interpretation of the result.

### Data analysis

Qualitative content analysis was used to analyse the interviews [22]. In the organizing phase the text was read and reread multiple times and meaning units correlating to the aim were chosen. The first and second author performed the analysis with the support of the last author. A reflection on the overall content was performed, where the aim was at the centre during the entirety of the analysis process. The meaning units (*n* = 212) were coded, and subcategories were retrieved by putting all of the codes in a document (see an example in Table 2). The different codes were assigned different colours depending on the focus. Codes with similar colour/content were grouped into preliminary categories. By moving back and forth between the meaning units, codes and preliminary categories, the authors identified the subcategories and links between them based on their similarities and differences. The six subcategories were abstracted into two generic categories.

**Table 2:** The process of the analysis

Meaning units	Open coding	Code	Subcategories	Generic categories
I am sitting, staring outside … that’s probably all the light we’ll get during our lunch hour …	Try to access the daylight that you can get	Daylight perceived to do good, physically	Having access to daylight	Difference in light
During lunch hour … the fact that you can look outside and see the daylight. I think it has a physical affect on me …	Try to access the daylight that you can get		Having access to daylight	Difference in light
… but it was hard at first, to not be able to see the light or get any daylight on your skin …	Very difficult to have limited access to light	Want to be able to look outside	Access to the surrounding world	Contact with the outer world

## Results

The result was presented using two generic categories and six subcategories. The generic category *Difference in light* consisted of the following subcategories: sensation of light, having access to daylight, and working in darkness. The generic category *Contact with the outer world* contained: Access to the surrounding world, , the sensation of seclusion, and the ability to choose.

### Difference in light

The sensation of light varies and affects the operating room nurses´ perceived health. The operating rooms did not have access to windows and daylight, thereby providing a limited experience in working in daylight. Daylight is not the only factor influencing the operating room nurse during surgery; available lighting in the operating room is another factor.

#### Sensation of light

Operating room nurses experienced a difference in light and daylight. Daylight provided a different feeling, which the electric light from the operating lamps could not replace. The positive feeling that accesses to light provided was difficult to explain, it was simply present. They also experienced a great difference in the light indoors compared to outdoors. During outdoor activities they had the combination of fresh air and daylight in greater amounts and perceived the ability to go outside to provide relief.

Daylight openings provided a deeper meaning in and of itself; they could look outside and become aware of their surroundings. Windows provided opportunities to look outside and have a different sense of time. The perception of time was lost during their time spent in the operating room, making the windows outside of the operating room seem even more important. As the nurses performed specific procedures in darkness the sensation of lack of perception of time or season was increased, it did not matter if this occurred during night or day.

*“This season [winter] is hard in that way, the fact that it’s so dark. That you don’t get the sunlight. Real light in a way”* Focus group 3.

#### Having access to daylight

Having access to daylight was considered important and something the nurses needed in order to experience wellbeing. Daylight affected their mood; it could lead to positive feelings such as a sensation of joy, but also increased awareness and energy. The operating room nurses experienced an increase in their wellbeing from spending time in daylight; life felt easier, and the lack of daylight provoked negative feelings.

*“I thought it was terribly difficult. God, am I supposed to work like this forever? It was really hard. I was frustrated and irritated and so on. It really had a negative effect on me. After a while you do get used to it”* Focus Group 2

Not having access to daylight during workdays contributed to fatigue. Daylight was seen as a contributing factor to health; not having access to daylight in the working environment, year in and year out, was considered to lead to psychological and physical effects. Increased periods of daylight, e.g., during spring or summer time, resulted in a decreased amount of sick leaves and could counteract mental effects such as depression as well as lead to increased energy.

The sunlight reached one of the staff areas creating an uncomfortable heat, to which the blinds were used. This caused frustration with the operating room nurses as it prevented them from looking outside. Lack of daylight lead to an inner stress and feeling bad for not having taken advantage of the daylight. The lack of daylight in the workplace created stress in the free time, as it resulted in “chasing of the light” as soon as they had a day off.

*“ … daylight is something necessary of which I have too little of and it’s almost as if when you’re having a day off you have to be mindful of it, almost chasing it … // … I feel that something I need is missing, and become stressed out to get it (during my day off) since we don’t get it during the week”* Focus Group 3

The operating room nurses perceived daylight as something necessary for their mental wellbeing as it provided a good sensation. It was even seen to improve the ability to perform, that they had the energy to improve their performance if they had access to daylight. The operating room nurse perceived that they became more sensitive as the years went by and that their eyes strained harder from working in darkness, and that coming out to the light gave a greater importance than previously held. It did, however, also affect the younger participants who had similar experiences. Artificial light could have positive effects as it would lead to increased alertness during on-calls at night.

#### Working in darkness

Light was shown to have a great significance in the operating rooms. In addition to the lack of daylight, the operating room nurses performed certain procedures in darkness. The advancement of surgical techniques has resulted in more procedures performed where everything is seen on screens. This requires darkness in the operating room to enhance visual sight, often during long periods of time. To work in darkness was perceived as stressful on the body and demanded mental preparedness before the surgery started.

*“ … you sort of brace yourself. This is what it is, alright let’s do this, this is the way it is now, we’re gonna have to stand in this darkness, it’s a matter of staying awake … ”* Focus Group 1

The physical strains that emerged affected, among other things, eyesight where the eyes struggled to focus, which resulted in headaches. Some described the experience as going into hibernation, which contributed to the feeling of decreased ability to perform, despite the fact that the procedure demanded a lot of focus. The feeling was enhanced during periods where the operating room nurse had a more passive role. They perceived it easier to work in light because working in darkness affected their ability to concentrate and this caused feelings of not feeling well. The frequency of working in darkness also affected the experience.

### Contact with the outer world

It was difficult to access the daylight situation during the workday, which resulted in a feeling of being locked in. The ability to look out a window at the workplace relieved this feeling. They thrived when they had the ability to choose.

#### Access to the surrounding world

The experience of the operating room nurses was that light and the ability to look outside during a workday was important. It provided a sense of freedom and was seen as having a positive effect on one’s wellbeing and giving access to the surrounding world. The placement of windows and what was located outside the window was considered important to how the operating room nurse experienced the situation.

*“even if we have windows on the entire wall of the break room, it’s still dark in there. So even if the sun is shining, it’s hard to see since you can’t really see the sky, the sun doesn’t reach us, it doesn’t really get to us down here”.* Focus Group 3

The ability to see the sky was important and diminished the need of going outside during the day. Being able to see the weather outside, whether the sun was shining could even offer social aspects as it became the conversational topic of the day. If the operating room nurse were able to partake in the report of weather, it leads to a sensation of missing part of the day as they conversed with family members or others. The weather outside also affects the wellbeing of the person. Seeing the sun through a window result in the mood of the co-workers improving. Being able to look outside and see the weather, season, and time of day contributed to a feeling of contact with the outer world and decreased the feeling of being locked in.

#### The sensation of seclusion

Having access to windows seemed to be a part of the structure of a room, according to the nurses. A room with four walls and a few doors but no windows provided a feeling of “being in a bunker”. There were so-called light courts located at the workplace, however, the ability to access those meant a process for the staff in that they had to change clothes before and after going outside. This lead to them staying indoors. The dressing rooms were located in the basement, making it more difficult, and taking more time. The light courts were described as:

*“ … you can’t see the sky anyways … // … but you see a relatively closed room with daylight”* Focus Group 4

The lack of windows was perceived as depressing. Although the sensation of getting used to the environment was also described, that they forgot the light when they could not see it. The nurses had previously had access to windows in one of the operating rooms, that was removed due to lack of space and was covered up. This provoked negative feelings as they primarily worked in that operating room during the weekends.

*“ … it was like a dream, being able to look outside. We did surgery in that operating room all the time during the weekends. I mean, just being able to see “oh” (happy vocalisation) the sun breaks through the clouds and even if it was for just two seconds, that was sort of enough”.* Focus Group 1

While the operating room still had the window, it was also portrayed with pride; if visitors came or future colleagues visited, that operating room was always displayed.

#### The ability to choose

The break room and the corridor outside the bathrooms had the most access to windows. The operating room nurses could choose whether to sit by a window and look out or sit the opposite way in the break rooms. Seeking access to daylight was deemed necessary as it was the only contact with the “outer world” they had during the day.

*“Then you’d like to place yourself by a window and look out and say: “So, this is what the weather is like today, or what we get today”.* Focus Group 1

The operating room nurses expressed the longing to go outside as it would provide more energy. They wished that specific changes could be made, like, among others, the ability to have a longer lunch break with the possibility of going outside to enjoy the daylight. Primarily during the winter months, as few opportunities to experience daylight existed after workhours. Even if there was a desire to get access to daylight, other important factors affecting wellbeing as well. The choice to not go out could be dictated by the need to rest and also by social aspects as the lunch provided the only opportunity to talk to their colleagues. However, the longing for daylight and the fresh air weigh heavier.

*“I was stressed in the beginning as I started this. But then I was like “no, I need to go outside, I can’t stand being inside … but I’d rather go outside and move around and get some daylight than sit down. Even if I also wanted to have time to sit for a few minutes.”* Focus Group 2

There was a desire to have more windows at the workplace, but the operating room nurses believed it to be difficult as the windows would be placed toward another building that would not allow them to see outside. Ceiling lights mimicking daylight were deemed to give better effect, while experiences from previous jobs included that those kinds of lights did not have the desired result. Other suggestions included improving the working environment with roofs over light gardens so that operating room nurses could sit there and see the daylight without a process of changing clothes.

## Discussion

The main findings in this study were that the operating room nurses experienced a sense of being locked in as a result of the difference in light and the lack of windows. Daylight in and of itself was deemed a positive factor for both physical and mental wellbeing in a different way compared to light fixtures. Working in darkness in the operating room was considered a negative influence on the wellbeing.

The results reveal that the need for daylight varies depending on the time of year. The operating room nurses experienced increased fatigue during wintertime when darkness is more pronounced. This can be correlated to the increased level of melatonin which can be seen during the winter and which exaggerates fatigue [11]. Working in the northern hemisphere with a limited amount of daylight has also proven to affect the mood of staff [13, 23], and is further increased by only working daytime, resulting in decreased hours of daylight [23].

It is of importance to have a good visual environment in order to experience wellbeing [6]. The operating room nurse may need to work in darkness or dim light in addition to limited access to daylight, which is considered to have a negative impact on mood. The present study has shown that working in darkness or dim light increases feelings of fatigue in staff. This relates to the work by Küller et al. [13] who reports that staff working indoors experience a decrease in mood as the lighting was considered too dark.

Thirty minutes of exposure to natural light with high levels has demonstrated to give a positive effect on mood that can last up to an hour [24]. In a study performed in a reception area, with access to windows and daylight, has showed to increase the frequency of laughter and social interactions compared to a windowless reception in the same ward [25]. This relates to this study results showing that operating room nurses experienced positive emotions, energy, and wellbeing as a result of daylight exposure. Being in an environment where one feels good can be seen as improving wellbeing. If a person experiences wellbeing in the workplace, this can improve performance. Daylight can be seen as a part of the physical environment, but according to Zadeh et al. [25] the workplace was improved when the sun could be seen through a window.

Access to a window with a view and the surroundings outside the window affected the operating room nurses experience of wellbeing and increased the sense of freedom, control and contact with the outer world. The surrounding environment of the workplace has proven to be an important factor [26] and is negatively affected by frosted glass, bushes, or buildings in close proximity. Access to a window and a viewpoint seemed to be the most important environmental factor when rebuilding a hospital [27]. It could be interpreted such that staff will adjust to their environment and not consider the meaning of daylight until they have been given access or lost previous access to daylight, which was also seen in the present study.

Working at a surgery ward in darkness or dim lighting appeared to increase fatigue and decrease the level of concentration for the operating room nurses. Whether this affected patient safety was not established in this study. However, Booker and Roseman [28] showed that limited access to daylight decreased the ability to perform and deal with stress, especially during the darker months of the year, which was deemed to increase the risk of wrongly medicating the patient. Decreased ability to concentrate can be related to a lack of patient safety [16]. As patient safety is an important question globally, this should result in interventions to improve the same. The care provided should be done in a, for the patient, as safe manner as possible, where unwarranted damage from care is avoided [29].

The Swedish Work Environment Authority [30] advocated for the presence of windows in the workplace due to the positive relation between access to daylight and wellbeing and ability to perform. Daylight allows for an accommodation of the diurnal rhythm, where people become more energetic as well as lead to an increase in wellbeing. A good work environment is important to operating room nurses as it can decrease stress as well as increase the surgical outcome [31].

### Limitations of the study

By presenting the method and design as detailed as possible, the authors facilitate the reader to partake in the study and evaluate it’s credibility [20]. Another strength of the study is that it includes a homogenous group, as participants are more prone to share their viewpoints if they have a similar background. The fact that the operating room nurses came from varied orientations within elective surgery, as well as that there were no exclusion criteria, provided an increased variation. This increases the credibility of the sample as well as the transferability of the results [32]. Using our own question-guide (Table 1) could affect the credibility of the study [21]. A test-interview was conducted to test whether the questions were comprehensible. This increases the trustworthiness and credibility of the result. Recording the interviews assured the result as this could avoid the notes or memory of the writers would impact the result [20], which increases the trustworthiness of the study. As the interviews were done in February when the staff of the surgical ward had limited access to daylight during the entire winter may have affected the trustworthiness of the study [32].

## Conclusion

This study shows that daylight is important for the wellbeing of the staff in the health care context. Daylight leads to energy and increased wellbeing. Since wellbeing facilitates reflection and complex thinking, it is not just the wellbeing of the staff that is affected, but also the safety of the patient. Absence of daylight created an inner stress in the operating room nurses, resulting in wrongly medicating the patient as well as affecting how the operating room nurse thrived in their profession. Modern surgery tends to be conducted more and more in darkness. This affects the physical as well as the mental wellbeing, resulting in increased fatigue, decreased ability to concentrate and also affecting eyesight. Lastly, the ability to look outside can decrease the feeling of being locked inside in the context of the surgery ward. Windows can directly correlate to increased wellbeing at the workplace. Increasing the wellbeing for this target group, which has a central role in every surgery performed, ought to be a prioritized issue.

### Relevance for clinical practice

This study adds to the understanding of the operating room nurse’s work environment and proves that daylight is a contributing factor that can affect the workplace’s wellbeing. An awareness of how the work environment can be designed can enhance the wellbeing of the staff and increase their ability to perform. Making the health care organisation conscious about how the operating room environment affects operating room nurses can lead to positive effects for both staff and patients. A clinical implication for workplaces with limited access to daylight could partly be compensated by elongating the lunch break, allowing the staff more time to get outside. The staff should also participate with advice and wishes in the case of rebuilding a surgery ward.

## Data Availability

The dataset generated and/or analysed during the current study is not publicly available, to preserve anonymity and integrity of the participants but is available on reasonable request via the corresponding author.

## Abbreviation

COREQ: Consolidated criteria for reporting qualitative research
